# Developing Electron Microscopy Tools for Profiling Plasma Lipoproteins Using Methyl Cellulose Embedment, Machine Learning and Immunodetection of Apolipoprotein B and Apolipoprotein(a)

**DOI:** 10.3390/ijms21176373

**Published:** 2020-09-02

**Authors:** Yvonne Giesecke, Samuel Soete, Katarzyna MacKinnon, Thanasis Tsiaras, Madeline Ward, Mohammed Althobaiti, Tamas Suveges, James E. Lucocq, Stephen J. McKenna, John M. Lucocq

**Affiliations:** 1 Structural Cell Biology Group, School of Medicine, University of St Andrews, North Haugh, St Andrews KY16 9TF, UK; cyg@st-andrews.ac.uk (Y.G.); ss366@st-andrews.ac.uk (S.S.); madeline.ward@student.manchester.ac.uk (M.W.); ma259@st-andrews.ac.uk (M.A.); 2 CVIP, School of Science and Engineering, University of Dundee, Dundee DD1 4HN, UK; k.w.mackinnon@dundee.ac.uk (K.M.); tsiarasa@hotmail.com (T.T.); t.suveges@dundee.ac.uk (T.S.); s.j.z.mckenna@dundee.ac.uk (S.J.M.); 3 Department of Orthopaedics, Ninewells Hospital, James Arrott Drive, Dundee DD1 9SY, UK; james.lucocq@nhs.net

**Keywords:** lipoproteins, nanoparticles, low-density lipoproteins, apolipoprotein B, apolipoprotein(a), electron microscopy, cardiovascular disease, machine learning

## Abstract

Plasma lipoproteins are important carriers of cholesterol and have been linked strongly to cardiovascular disease (CVD). Our study aimed to achieve fine-grained measurements of lipoprotein subpopulations such as low-density lipoprotein (LDL), lipoprotein(a) (Lp(a), or remnant lipoproteins (RLP) using electron microscopy combined with machine learning tools from microliter samples of human plasma. In the reported method, lipoproteins were absorbed onto electron microscopy (EM) support films from diluted plasma and embedded in thin films of methyl cellulose (MC) containing mixed metal stains, providing intense edge contrast. The results show that LPs have a continuous frequency distribution of sizes, extending from LDL (> 15 nm) to intermediate density lipoprotein (IDL) and very low-density lipoproteins (VLDL). Furthermore, mixed metal staining produces striking “positive” contrast of specific antibodies attached to lipoproteins providing quantitative data on apolipoprotein(a)-positive Lp(a) or apolipoprotein B (ApoB)-positive particles. To enable automatic particle characterization, we also demonstrated efficient segmentation of lipoprotein particles using deep learning software characterized by a *Mask Region-based Convolutional Neural Networks (R-CNN)* architecture with transfer learning. In future, EM and machine learning could be combined with microarray deposition and automated imaging for higher throughput quantitation of lipoproteins associated with CVD risk.

## 1. Introduction

Plasma lipoprotein (LP) nanoparticles are composed of cholesterol esters, dietary triacylglycerols, free cholesterol, phospholipids, and apolipoproteins and comprise a wide spectrum of sizes, ranging from chylomicrons (75–1200 nm) through low-density lipoproteins (LDL, 18–25 nm) to high-density lipoprotein (HDL, 5–12 nm) [1,2]. Although LDL has long been recognized as a primary associated risk factor for cardiovascular disease (CVD) [1,3,4,5], a lengthening list of LP subpopulations is of interest in the pathogenesis of arterial disease. These include small dense LDL (sdLDL) [6,7], lipoprotein (a) (Lp(a)) [8,9], and remnant lipoproteins (RLPs). sdLDL (18.0–20.5 nm) [10] is proposed to be more atherogenic than LDL by means of its small size, which could enhance penetration into the vessel wall. It also has a longer plasma half-life due to a lower affinity for the LDL receptor and a decreased resistance to oxidation. Lp(a) (21 nm) [11] shares many characteristics with LDL and elevated levels of Lp(a) predispose to a variety of cardiovascular diseases. Lp(a) levels are largely determined by genetics, are resistant to lifestyle changes or traditional lipid-lowering drugs and are difficult to assay because of variations in size/composition [12,13,14,15,16,17,18]. Remnant lipoproteins represent chylomicrons or VLDLs (very low-density lipoproteins) that are partially catabolized to become smaller and denser [1] and are thought to be more strongly atherogenic than their “parent” triglyceride-rich lipoproteins [19,20,21]. Assays for sensing these diverse biomarkers are being developed [22] but, as yet, a fine-grained and comprehensive profiling of sizes, numbers, and/or molecular composition across all such LP species [23] is not available.

One solution is to reveal the whole distribution of lipoproteins as nanoparticles by direct imaging, and here we investigated development of electron microscopy as a primary tool. Currently there is a range of methods for quantifying lipoprotein nanoparticles. Most rely on biophysical properties and include nuclear magnetic resonance (NMR) [24], nanoparticle tracking analysis [25], flow cytometry [26], dynamic light scattering [27], small angle X-ray, and resistive pulse sensing. NMR is an indirect method that responds to methylation signals and can be used to characterize LDL profiles from human plasma, but the estimates of particle size differ from those determined in gradient gel electrophoresis, despite using the same samples [28,29]. Also, NMR has only rarely been validated using benchmark electron microscopy methods [24]. Nanoparticle tracking analysis is widely used and based on the principles of light scattering and Brownian motion. However, nanoparticle tracking analysis is known to be susceptible to lab-to-lab variation, detects only larger lipoproteins [30], and particle selection is difficult to randomize [25]. Importantly, recent data suggest that nanoparticle tracking analysis cannot differentiate between extracellular vesicles and LPs [2,31,32]. Dynamic light scattering also uses Brownian motion, and measures fluctuations in intensity of light scattered by nanoparticles [27], but again sizing of particles under 30 nm and differentiation of large particles from aggregates is problematic. Small angle X-ray and resistive pulse sensing remain under-investigated for lipoprotein particles but require expensive apparatus although differential ion mobility analysis reveals a more continuous distribution of particle sizes [33]. Collectively, the largely indirect nature of these methods makes them prone to systematic errors, unable to differentiate nanoparticle types such as extracellular vesicles/LPs, and insensitive to aggregation state of the particles [34].

Transmission electron microscopy is an established method that provides enough resolution to image an extensive range of lipoprotein particles and could be used in routine quantification if adapted to high throughput. The two main transmission electron microscopy (EM) methods currently in use are cryo-electron microscopy (cryo-EM) and negative staining. Cryo-EM provides benchmark imaging of minimally denatured particles embedded in vitrified water but is not suitable for quantification because the images are of low contrast, instruments are difficult to maintain, and sampling of particles is compromised by frequent artefacts. On the other hand, negative staining, which is based on deposition of heavy metals around the particles, is more reproducible and rapid than cryo-EM, allowing better sampling of particles. While there are some problems of inconsistent contrasting and collapse of soft biological particles, improvements have been made by adding hydrophilic embedding media such as methyl cellulose containing mixtures of metals for contrasting. Such embedment helps to support the particles and provides even and consistent films, allowing appropriate sampling and accurate quantification. A further, and recent, modification incorporates a countable calibrant in the support film, providing quantitative readouts of particle concentration [34].

In this report we developed transmission electron microscopy for quantifying lipoproteins in human plasma. The method provides contrasting and imaging that allows quantification of LPs directly from microliters of non-purified human plasma. It detects a continuous size distribution of LPs from the smallest LDL particles through to VLDL and can be combined with antibody contrasting to identify subpopulations such as Lp(a). Finally, as a prelude to higher throughput, we established automated recognition for characterization of LPs using machine learning. Together, these studies demonstrated the feasibility of visualisation, recognition, and quantification of multiple species of LPs from human plasma using electron microscopy. They represent a step toward higher throughput measurements on LPs from microliter samples of human plasma.

## 2. Results

### 2.1. Contrasting and Optimization of Methylcellulose (MC) Film Thickness

**Figure 1:**
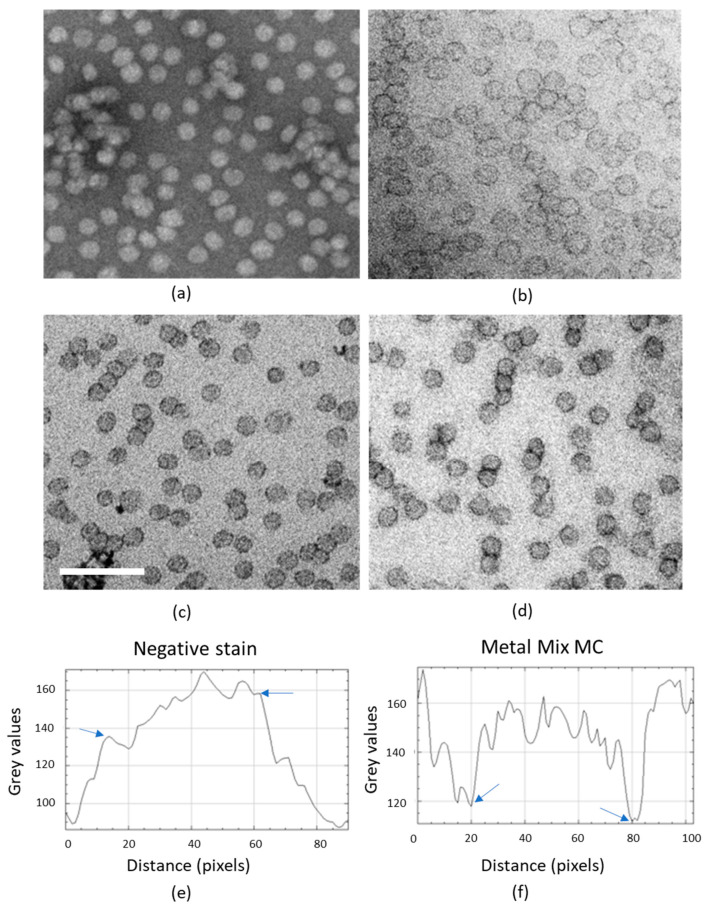

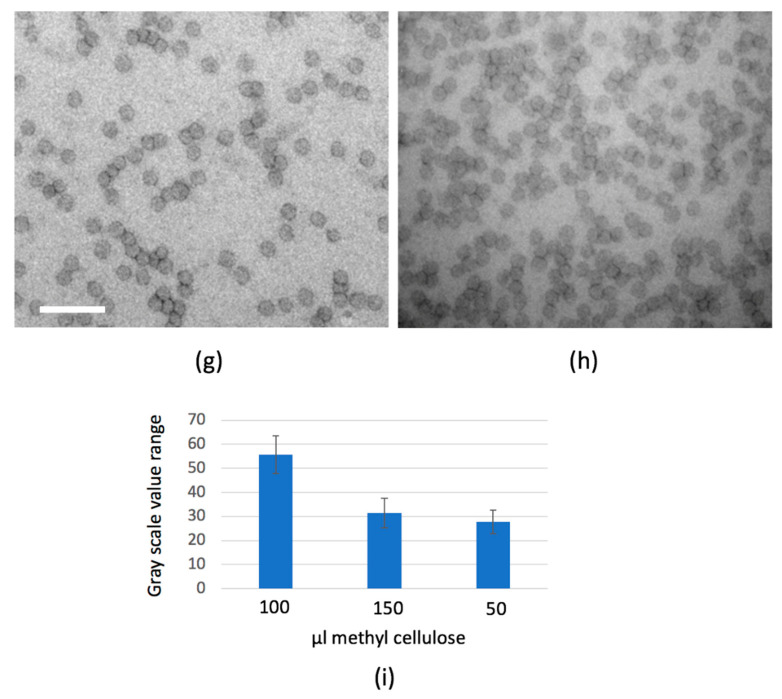
Optimization of mixed metal MC staining on purified low-density lipoprotein (LDL). (**a**) Negative stain using uranyl acetate (UA), see Asadi et al., [36]. (**b**) Heavy metal mix in thick methyl cellulose (MC), (800 µL MC, 100 µL UA, 25 µL sodium silicostungstate (STA)). (**c**) and (**d**) Heavy metal mix in thin MC film. (**c**) 100 µL MC, 25 µL UA, 100 µL STA, and 875 µL H_2_O, and (**d**) 100 µL MC, 50 µL UA, 25 µL STA, and 925 µL H_2_O. Concentrations as described in Materials and Methods. In negative stain (**a**) edges are fuzzy while (**d**) shows positive contrast at the particle edges. (**e**,**f**) Show profile intensity plots from ImageJ, indicating distinct transitions at the inner surface of the particle edge density for the heavy metal mix (metal mix MC; **f**, arrows). In the negative stain, edge transitions (likely at the outer surface) are more difficult to define (**e**, arrows). (**g**–**i**) Show further testing of film thickness and staining protocol for lipoproteins in a purified LDL preparation using heavy metal mix in thin MC films. (**g**) 50 µL MC, 50 µL UA, 25 µL STA and 975 µL H_2_O; (**h**) 150 µL MC, 50 µL UA, 25 µL STA and 875 µL H_2_O. (**i**) Contrast expressed as the range in grey scale values through particle edge to background as determined in ImageJ. *n* = 10 particles in each case; 50, 100, or 150 µL of 2% MC in a total volume 1100 µL, containing 50 µL UA, 25 µL STA, and H_2_O. Error bars are standard deviations. Scale bars 100 nm (in (**c**) for (**a**–**d**) and in (**g**) for (**g**,**h**).

Optimization of contrast for staining lipoproteins was achieved using a commercial low-density lipoprotein (LDL) preparation. LDL was diluted either in water or in phosphate buffered saline (PBS) and absorbed onto plastic-coated electron microscopy (EM) support grids. In the workup, we determined that 30 min to 1 h was the best time period for reproducible absorption of nanoparticles from the test suspension (not shown). We next tested a range of electron stains, including conventional negative stain and stains containing MC as an embedding medium support [35]. MC supports the particles, and the thickness can be varied according to the starting volume before drying down inside a loop of tungsten metal wire [35]. Conventional negative stain using uranyl acetate (UA) produced expected negative contrast with low-density particle interior and an indistinct edge (Figure 1a). A combination of MC and UA produced delicate positive contrast with a stained particle periphery but the contrast between the edge and background was relatively low (not shown). Enhancement of edge contrast was observed after including sodium silicotungstate (STA) in the staining mixture (Figure 1b, mixed metal staining). This modification has previously been shown to enhance contrast on a range of nanoparticles such as lipid-rich nanodiscs [36].

We investigated whether a reduction in contrasting-film thickness, achieved by reducing the starting concentration of methylcellulose, could further improve edge contrast of LDL. Low-density lipoproteins measure approximately 20 nm in diameter and are 12 nm in thickness and should be best contrasted when film thickness approximates to the size of the particles. Conventional amounts of MC administered by means of drying down within wire loops are thought to produce films with a thickness capable of supporting a 110-nm ultrathin cryo-section [35]. We, therefore, reduced methyl cellulose to 1/8 of that used in Figure 1b for contrasting LDL. This produced striking improvement in contrast, especially after mixed metal staining (UA plus STA) (compare Figure 1b with Figure 1c,d). Quantitative analysis of contrast at the edge of the particles confirmed clearer gradients with MC UA/STA mix compared to negative stain (Figure 1e,f).

An additional advantage of thinner MC films is that flattened spheroidal particles such as LDL and nanodiscs tend to orient *en face* to the film, rather than taking up a range of orientations [34,36,37]. This effect makes the particles present a more homogeneous-sized profile to the electron beam. Accordingly, we found the ratio of major to minor axis was 1.42 (23.28 nm/16.28 nm) for thicker MC films and 1.29 (23.81 nm/18.33 nm) for thinner MC or 1.28 (23.59 nm/18.47 nm) for negative stain lacking MC (*n* = 100 particles in each case, measured across the particles manually between inner edges of positive contrast).

We next performed a more fine-grained analysis of the effects of MC amounts on LDL contrast (Figure 1g–i), doubling or halving MC used in Figure 1. Neither modification improved the contrast as measured by maximum/minimum differences across the particle edge. With the knowledge that thinner MC films produced better contrast and less asymmetric profile data, we selected the following combination—100 µL MC, 50 µL UA, 25 µL STA in a total volume of 1100 µL (standard mixed metal MC)—for contrasting lipoproteins in this study.

**Figure 2:**
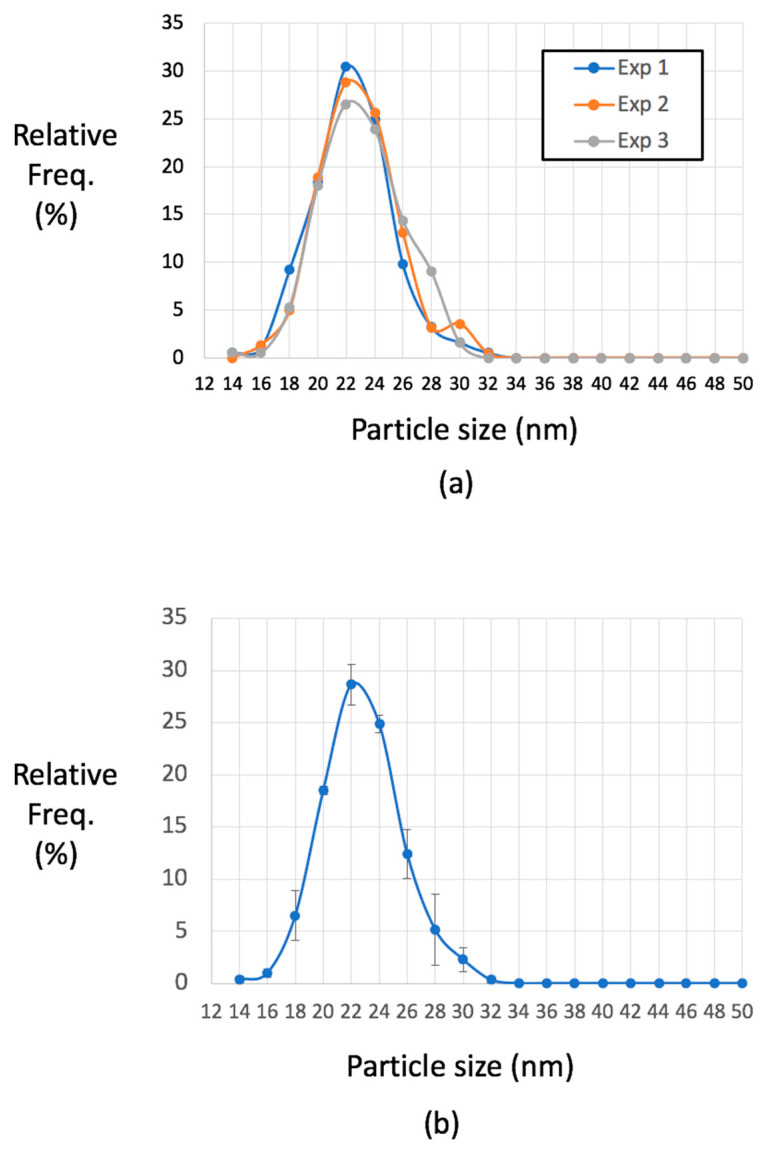
Reproducibility of particle analysis from a purified LDL preparation. In (**a**), three samples from the same LDL preparation were processed using the standard mixed metal MC and horizontal calliper distance measured in 185, 222, and 188 particles. (**b**) Means +/−SD (x axis labels refer to lower limit of 2-nm bins). No significant difference between these distributions with means 23.41, 23.82, 24.00; Kruskal-Wallis test statistic H = 5.778, df 2, *p* = 0.056. Data are expressed as the relative frequency of particle number for each particle size category expressed as a percentage of total particles analyzed (Relative Freq.%).

The reproducibility of particle size distributions was studied using a commercial LDL preparation. For simplicity and speed in assessing sizes, we measured particles in a horizontal calliper direction across each particle. This ensured a random direction with respect to each particle profile. This measurement was used throughout the rest of this report. Three independent experiments were used to assess concordance between measurements of LDL (Figure 2). Mean measurements for the three samples were 23.41, 23.82, 24.00 nm (mean = 23.74 nm, SD = 1.27, coefficient of variation (CV) = 2.28%; *n* = 185, 222, and 188, respectively; Kruskal–Wallis test was not significant, see legend to Figure 2). LDL particles have been reported as discoid in shape, measuring 21.4 by 12.1 nm [38] and so we computed corrected values for mean radius of a corresponding spheroid (20.01, 20.36, and 20.52 nm (mean = 20.3 nm, SD = 0.26)) to facilitate comparison to methods that are sensitive to mean rotational diameters.

### 2.2. Antibody-Binding Studies

Lipoproteins have well-defined lipid and protein compositions and it should be possible to combine mixed metal MC with antibody labelling experiments for identification of subpopulations of LPs. Thus, LDL contains apolipoprotein B100 (ApoB100) while apolipoprotein(a) is specific for Lipoprotein (a)(Lp(a)) [1]. We, therefore, evaluated different approaches for imaging antibody binding.

**Figure 3:**
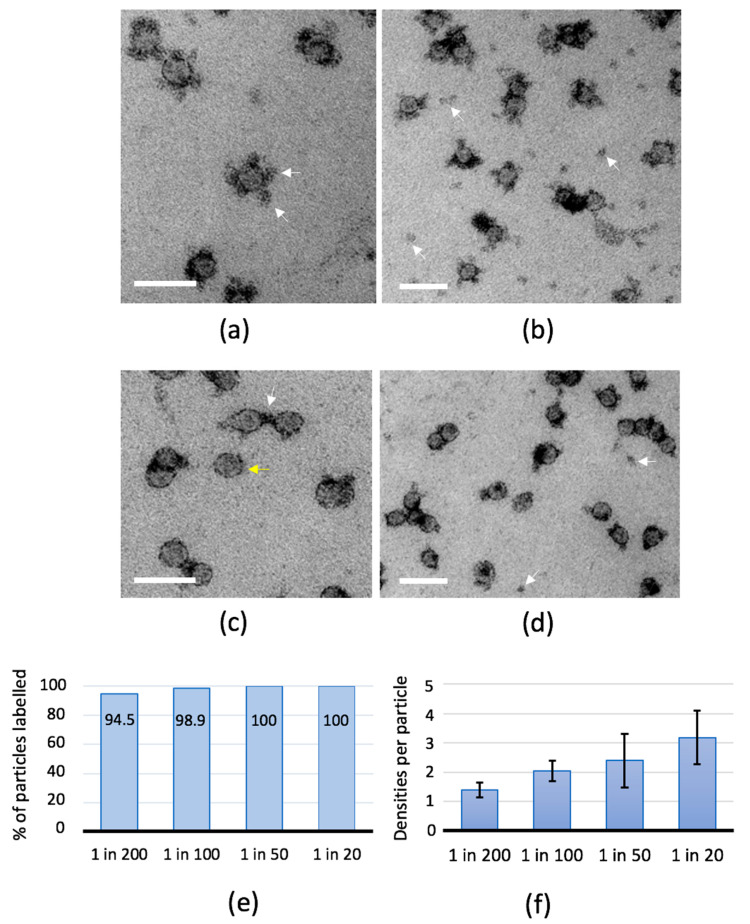
Antibody labelling of purified LDL. LDL adsorbed to support films was labelled using polyclonal antibodies raised against apolipoprotein B (ApoB) and contrasted with mixed metal MC. (**a**,**b**) 1/20 dilution and (**c**,**d**) 1/200 dilution. In (**a**) (high-magnification) the antibody densities surround LDL particles (arrows) and, in (**b**) (low-magnification) the particles are surrounded by multiple antibody densities with abundant contrasted features in between, likely representing unbound antibody. In (**c**), some LDL particles have no associated antibody density (yellow arrow) and others appear to have bridges of contrast spanning between the two particles (white arrow). In (**d**) the low-magnification overview shows fewer isolated densities (putative unbound antibody, white arrows) than are seen in (**b**). Scale bars 50 nm. Increasing the concentration of antibody maximizes the percentage of LDL particles that are labeled (**e**) and also increases the number of densities associated with each LP (error bars in (**f**) represent SD; >100 particles analyzed in each case).

We attempted immunogold [39] labelling of ApoB100 on purified LDL using polyclonal antibodies raised against ApoB. The antibody is expected to bind to multiple epitopes on ApoB100 in LDL, IDL, and VLDL and also to the apolipoprotein B48 in chylomicrons. However, this approach produced gold labelling on no more than 5% of the total particles, indicating the labelling efficiency was low, perhaps due to steric factors. Given the improvements in edge contrast of LPs and positive contrast of plasma proteins, we next assessed whether mixed metal MC staining could reveal primary anti-ApoB antibody bound at the periphery of the particles in the absence of the secondary gold label. This produced striking contrast of heavily stained “blobs” of stain surrounding the LDL particles (Figure 3a–d). These “blobs” measured 13.37 nm, SD = 3.55, *n* = 70 (range 7–22 nm), which corresponded to published values for antibodies bound to LDL or very low-density lipoprotein (VLDL) [40,41]. The blobs were found only in presence of the primary antibody and increasing the concentration of antibody increased their number from approximately 1 to 3 per particle, labelling 100% of the particles (Figure 3e,f). We concluded that application of antibody combined with mixed metal MC produces labelling of all the particles in the purified LDL preparation and appears to be a useful approach for labelling mixed populations of ApoB100 containing LPs (see below for labelling Lp(a) in human plasma).

**Figure 4:**
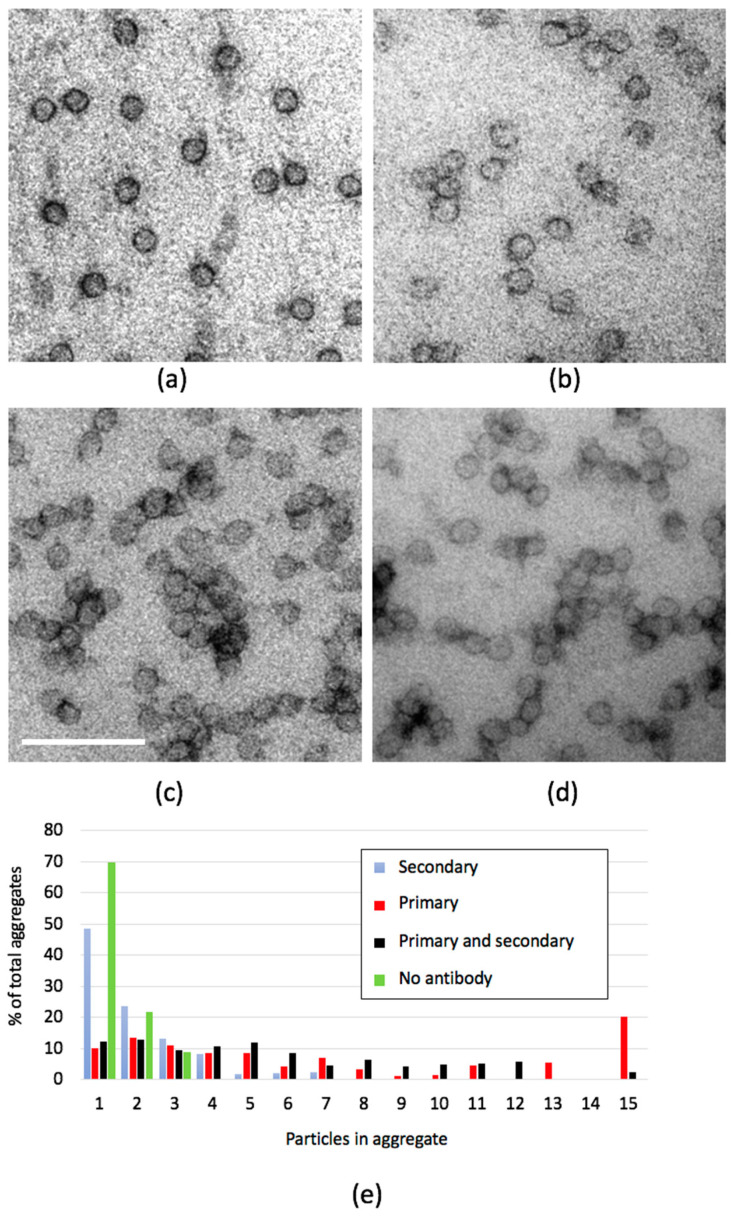
Antibody-induced aggregation of LDL on electron microscopy (EM) support films. This strategy, an alternative to direct contrasting, aims at detecting antigens by crosslinking particles with specific antibodies in an approach that is akin to hemagglutination. LDL particles were adsorbed to the support film and exposed to anti-ApoB polyclonal antibodies. (**a**) No antibody, (**b**) secondary antibody alone, (**c**) primary antibody, and (**d**) primary followed by secondary antibody. Notice aggregation of LDL appears similar in extent in (**c**) and (**d**). Scale bar in (**c**) 50 nm, for (**a**–**d**). (**e**) Quantification of the aggregation under same conditions as illustrated in (**a**–**d**). Distributions for control (no-antibody) and secondary only (secondary) are similar. Both primary alone (primary) and primary plus secondary (primary and secondary) conditions lack single particles and two-particle aggregates and contain more large aggregates. primary vs. secondary, chi square = 64.29, df 3, *p* < 0.001; primary vs. no antibody, chi square = 53.89, df 3, *p* < 0.001; no antibody vs secondary, chi square = 1.81, df 3, *p* < 0.2.

Another way to assess the presence of antigens in LPs is by antibody-induced aggregation, in an approach similar to that used in hemagglutination assays. We tested whether the anti-ApoB antibodies would induce the formation of LP aggregates in solution, prior to adsorption to the grid support and imaging. While this approach produced antibody-dependent aggregation, the adsorbed aggregates contained overlapping particles, which could not be easily evaluated by EM imaging. It is well known that some negative stains cause rouleaux formation of nanoparticles during negative staining on a grid support, which implies the particles are mobile. We took advantage of this movement to more easily monitor aggregation of particles in the plane of the support. Indeed, we found that specific primary anti-ApoB antibodies but not non-specific (secondary) antibodies caused aggregation (Figure 4) when applied after adsorption of purified LDL. The change in frequency distribution of aggregates was highly significant statistically (see Figure 4 legend) with 51% of the particles redistributing from the single particle pool or smallest aggregates (2 and 3 particles) to larger aggregates after exposure to the specific antibody. In the presence of the blocking agent fish skin gelatin, substantial aggregation did not occur, suggesting that aggregation is blocked when the proteins are pre-bound to the EM support film. This technique, therefore, shows promise as a method for identifying particles that contain specific antigens in a mixed population of LPs, but is not investigated further in this report.

### 2.3. Optimizing Visualisation of Lipoprotein Particles from Human Plasma

**Figure 5:**
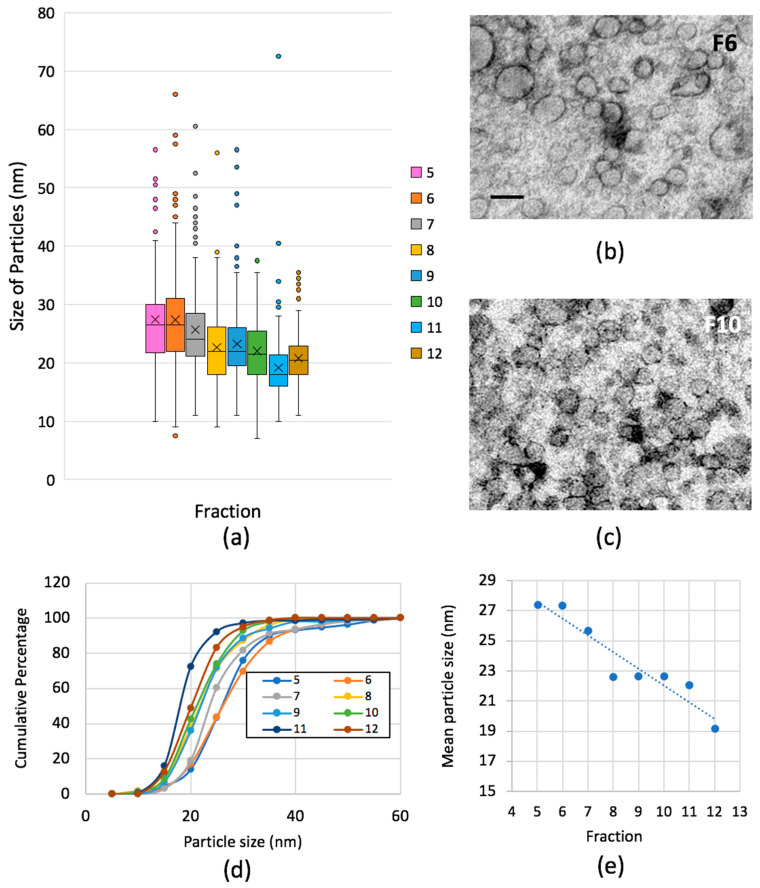
Lipoprotein particles in gel filtration fractions from human plasma. Fraction 4 and fraction 13 showed no detectable vesicles/LPs. (**a**) Black horizontal lines are mean size, boxes and error bars are quartiles above and below mean excluding outliers (dots); x marks median. (**b**) and (**c**) illustrate LPs found in fractions 6 (F6) and 10 (F10), respectively. (**d**) Comparison of cumulative distributions across gel filtration fractions (grouped into 5-nm size ranges); Kolmogorov-Smirnov (KS) tests as follows: F5 vs. 6 not significant; for F5 vs. 7, 8, 9, 10, 11 or 12 *ps* = or < 0.001. (**e**) Mean particle size decreased progressively across the fractions; y = −1.1122x + 33.162, R² = 0.8992. Scale bar 50 nm for images in both (**b**) and (**c**).

Our next step was to develop ways of imaging LPs from microliter samples of human plasma using mixed metal MC staining and then apply the antibody-binding technique for identifying Lp(a). Initially, we found absorption of neat plasma resulted in strong background densities that were interpreted as plasma proteins. One simple method for separating LPs from plasma proteins is size exclusion chromatography (SEC) [5], which has been used to separate lipoproteins and extracellular vesicles from plasma proteins [2,42,43]. We applied 150 µL of freshly thawed plasma to a commercially available Sepharose CL 2B filtration column (qEV single 70 nm, SP2, Izon Science, Oxford, UK) and assayed the output of protein (Figure S1) and lipoproteins using EM (see Figure 5). A wide range of LP particle sizes were found in the initial protein-poor fractions with the majority displaying regular circular profiles. In these early fractions, a small proportion of total profiles (<1/1000) were extremely large in size with slightly irregular or a collapsed appearance and were interpreted as extracellular vesicles. In fractions 5–9 LP edges were well contrasted but in fractions 9–12 numerous indistinct densities (consistent with adsorbed plasma proteins) interfered with imaging of LPs. The mean LP particle size reduced progressively through these fractions from 27.43 nm in fraction 5 to 20.80 nm in fraction 12 (Figure 5a,d,e). A decreasing number of large outliers, measuring 40 nm or more, were concentrated across fractions 5–9 with the distributions becoming statistically distinguishable (Kolmogorov-Smirnov (KS) test, Figure 5 legend). Further analysis showed fractions 5–8 had the largest proportions of particles between 20–40 nm while fractions 8–12, inclusive, had major populations between 15 and 20 nm. Protein was not detectable in fractions 1–10, while 0.11 and 0.48% of the total protein applied to the column was present in fractions 11 and 12, respectively. Increasing plasma proteins’ profiles made it difficult to characterize LP populations in the fractions 13 and upwards, so these protein-rich fractions were not investigated further by EM (see Figure S1).

**Figure 6:**
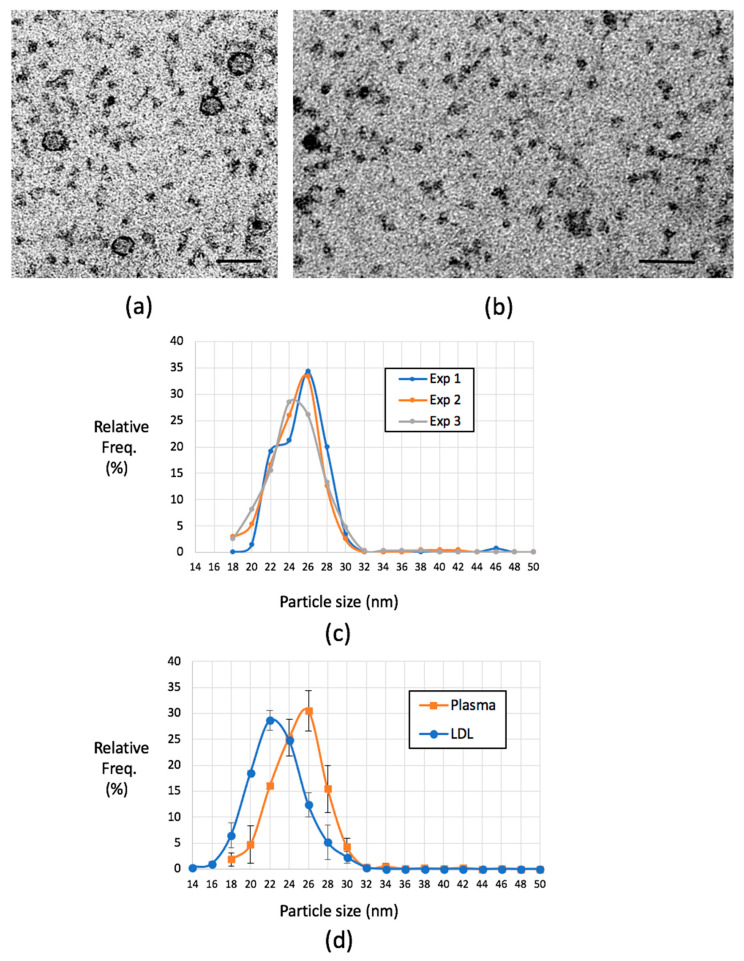
Visualisation of LPs in diluted human plasma. (**a**) Human plasma diluted 1 in 4000 in phosphate buffered saline (PBS). Clear lipoprotein particles with strong edge contrast are interspersed with putative plasma proteins also seen in (**b**). (**c**) Three samples of human plasma processed and quantified (>100 particles examined in each case). Distributions were not significantly different; Kruskal–Wallis test statistic H = 0.326, *p* = 0.8494. (**d**) Distribution of mean LP sizes compared to purified LDL data (error bars SDs). Scale bars in (**a**) and (**b**), 50 nm. In (**c**) and (**d**) data are expressed as the relative frequency of particle number for each particle size category expressed as a percentage of total particles analyzed (Relative Freq.%).

The failure of SEC to successfully separate LPs from plasma proteins prompted us to seek more straightforward methods of visualizing LPs from patient plasma samples. We attempted immuno-absorption of LPs using antibodies raised against ApoB that had been pre-bound to an EM support film, but this was not successful. A more straightforward approach was dilution of human plasma in buffer (PBS) at 1/2000, 1/3000, or 1/4000, followed by adsorption and contrasting with mixed metal MC. This procedure produced clear and reproducible images of LPs with spheroidal profiles and well-defined edge contrast. Smaller, less-distinct densities were present between the particles (Figure 6a,b). We next tested staining conditions across a range of mixed metal MC conditions and dilutions and found the standard mixed metal MC stain used for LDL provided optimal contrast. The resulting particle size distributions of LPs from 1/3000 dilutions of plasma samples are shown in Figure 6c,d (25.74, 25.75, and 26.42 nm (mean = 25.968 nm, SD = 0.392, CV 1.51, *n* = 3; no significant difference between the distributions by Kruskal–Wallis test, see legend to Figure 6). Visible LPs had clear linear contrast transitions at the edges and measured from approximately 14 nm upwards, which is a lower size range for LDL, through to larger particles in the size range of VLDL. A large number of less-distinct particles were present in the sub-14-nm range, which is likely to include plasma proteins and putative HDL [44].

As we previously observed with nanodiscs [36], thin films of mixed metal stain MC reduced the profile asymmetry so that the mean major–minor axes measurement ratio of adsorbed plasma LPs was 1.11 (SD = 0.096, *n* = 32), as compared to an expected ratio for LDL of 1.77 (major axis 21.4 ± 1.3 nm and minor axis 12.1 ± 1.1 nm [45]). This was consistent with the particles lying flat and presenting *en face* to the electron beam (note that this effect might not be relevant for larger, less asymmetric but angular VLDL particles [41]). Thus, our EM measurements tend to report maximal diameters, and so we applied a correction factor for the average diameter of an equivalent spheroid to facilitate comparison to results from other methodologies. A plot of particle size distribution compared to a previous LP classification [10] is shown in Figure S2. Thus, this analysis appears indicates the method detects particles across the full range, from very small LDL up to large VLDL. After correction, mean particle size of plasma LPs was 22.3 nm (SD = 0.333, CV 1.5%, *n* = 3) and the majority of the particles detected were in the LDL-size range (Figure S2).

### 2.4. Lp(a) Particles Identified Using Anti-Apolipoprotein(a) Antibodies

**Figure 7:**
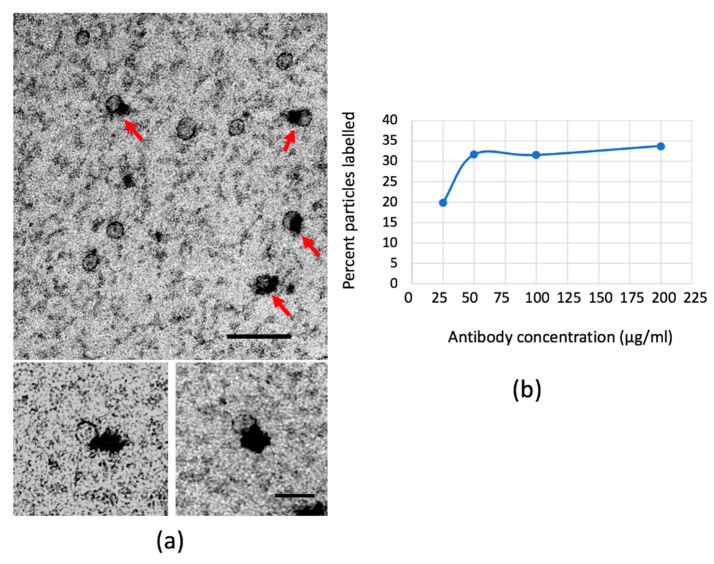

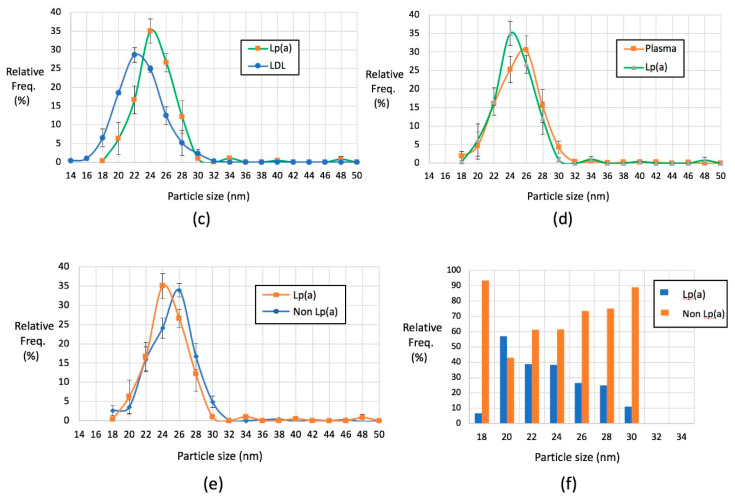
Lp(a) LPs identified using anti-apolipoprotein(a) antibodies. Human plasma adsorbed to EM supports and exposed to polyclonal anti-apolipoprotein(a) antibodies produce densities (arrows) associated with a subpopulation of LPs ((**a**) with details illustrated in two bottom panels; scale bars 100 nm and 50 nm, respectively). (**b**) The percentage of apolipoprotein(a)-positive particles plateaued with increasing antibody concentration. Frequency distribution of apolipoprotein(a)-positive particle sizes compared to purified human LDL (**c**); human plasma (**d**) and non-Lp(a) particles (**e**). (**f**) shows the percentage of total particles at each size category that are positive or negative for apolipoprotein (**a**). Error bars are SD from three experiments from the same sample of human plasma. >100 particles sampled in each case. In (**c**), (**d**) and (**e**), data represents the relative frequency of particle number for each particle size category expressed as a percentage of total particles analyzed (Relative Freq.%). In (**f**), Relative Freq.% represents the percentage of total particles found in each size category that were positive or negative for Lp(a).

The positive contrast provided by mixed metal staining provided an opportunity to use antibody labelling to identify the subpopulation of lipoprotein particles that comprise Lp(a), which are particles that harbor both ApoB and apoliprotein(a). Human plasma adsorbed at 1/3000 dilution was exposed to antibodies to apolipoprotein(a) and contrasted with mixed metal MC. A subpopulation of LPs became labelled with densities and the fraction of labelled particles stabilized with increasing antibody concentration, indicating saturation of binding sites (Figure 7a,b). Most particles were labelled with a single “hit” rather than the multiples seen with anti-ApoB. The distribution of Lp(a)-positive particle sizes peaked at 24 nm (larger than purified LDL; Figure 7c) and smaller than the peak obtained with whole plasma (Figure 7d). Interestingly, the particle size with the highest proportion of total particles that were positive for apolipoprotein(a) was at 20 nm (average particle size for Lp(a) mean 25.02 nm, SD = 1.158, CV 4.63%, *n = 3*). There was no detectable effect of freeze–thaw on the Lp(a) particle size distribution (Figure S3). The proportion of total plasma LPs that were apolipoprotein(a)-positive was 36.6% (SD = 1.42, CV 3.88%, *n* = 3).

### 2.5. Deep Learning Approach to Identifying Lipoproteins

Image quantification is slow and labor intensive and, in the case of lipoprotein particle analysis from patients, modifications to the workflow will be necessary to improve throughput. Key aspects include the incorporation of methods to automate and multiplex sample handling, preparation, and imaging. Another significant roadblock is recognition and quantification of the particles, which is currently done by applying eye–brain recognition to identify the structures of interest followed by image processing or stereological probes to quantify the particles.

To initiate steps toward higher throughput, we applied machine learning techniques for recognition of LPs. Recognition software was developed using a sample composed of purified commercial LDL particles embedded and contrasted using UA (without addition of STA). This provided image data with weaker positive edge contrast and more noise than obtained with the mixed metal MC. This made the recognition task more challenging. A development set of 37 images was used for model training and selection. The recognition software was then tested on two held-out test sets. Test set 1 had four images prepared similarly. Test set 2 had images prepared with the mixed metal MC, resulting in improved visibility of particle contours.

The 41 images contrasted using MC/UA alone were manually annotated under supervision of an experienced microscopist (J. M. Lucocq), who made corrections as necessary. Annotation involved delineating the boundary contours of each LDL particle, i.e., performing instance segmentation. Only those particles entirely located within the central 820 × 820 pixel-window of each image were annotated and analyzed. More than 4200 LDL particles were annotated. The number of instances in an image ranged from 22 to 168. Annotated particle size varied from 368 pixels^2^ to 13,164 pixels^2^ with median 1632 pixels^2^. (Taking the square root of the area as a linear measure of size, the range was 19.2 to 114.7 pixels with median 40.4 pixels).

Image augmentation, implemented using the imaging library, was used to increase the amount of annotated training data. Both nuclei detection pretraining and LDL instance segmentation training used augmentation by horizontal and vertical flip, rotation (90°, 180°, and 270°), brightening or darkening (multiplication by a random value between 0.5 and 1.5), and Gaussian blur. Experiments were also made with additional augmentations (additive Gaussian noise and sharpening) when training LDL instance segmentation.

The development dataset was divided into a 33-image training set and a four-image validation set. Validation set loss was used for early stopping with an upper limit of 50 training epochs. Four combinations of data augmentations and Region Proposed Network (RPN) anchor scale sets were tried and those with the most promising validation set performance were subsequently tested on the held-out test sets. Training took less than 2 h using an 8 GB Nvidia GTX1070 Graphic Processing Unit (GPU; the exact time depending on the parameters used).

Instance segmentation was evaluated within the central window of each image; only annotation masks that had at least 50% of their area within this window were used for evaluation. Particle detection rate (the proportion of particles that were successfully detected), the number of false-positive detections per 100 detections, and the median average precision (mAP) were used to assess performance. The latter used the mAP for Intersection over Union (IoU) from 0.50 to 0.95 with a step size of 0.05, where IoU denotes the intersection over union measure of overlap between an instance segmentation and its ground-truth segmentation.

**Figure 8:**
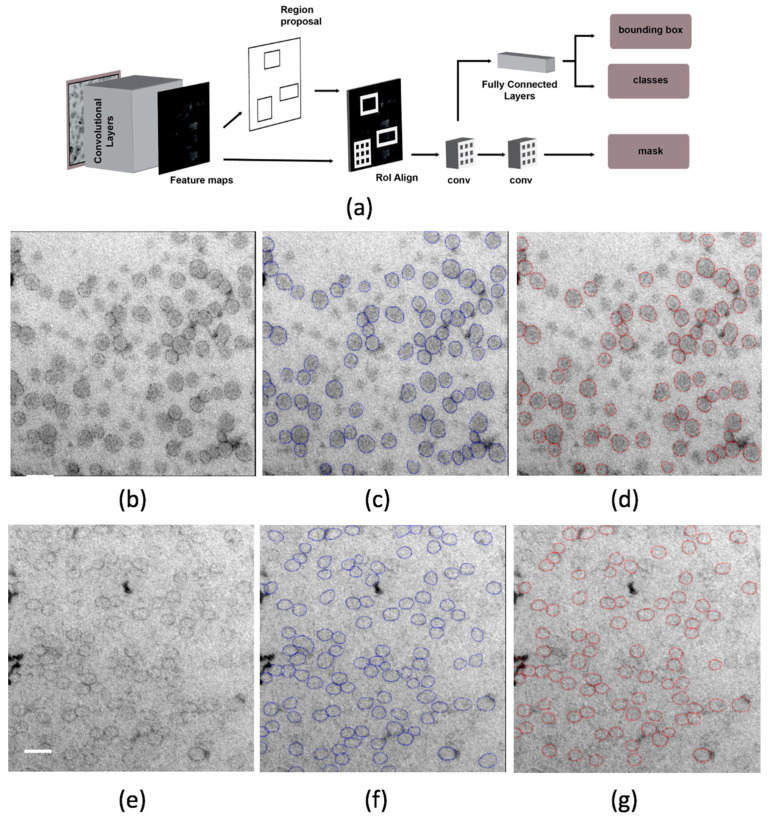

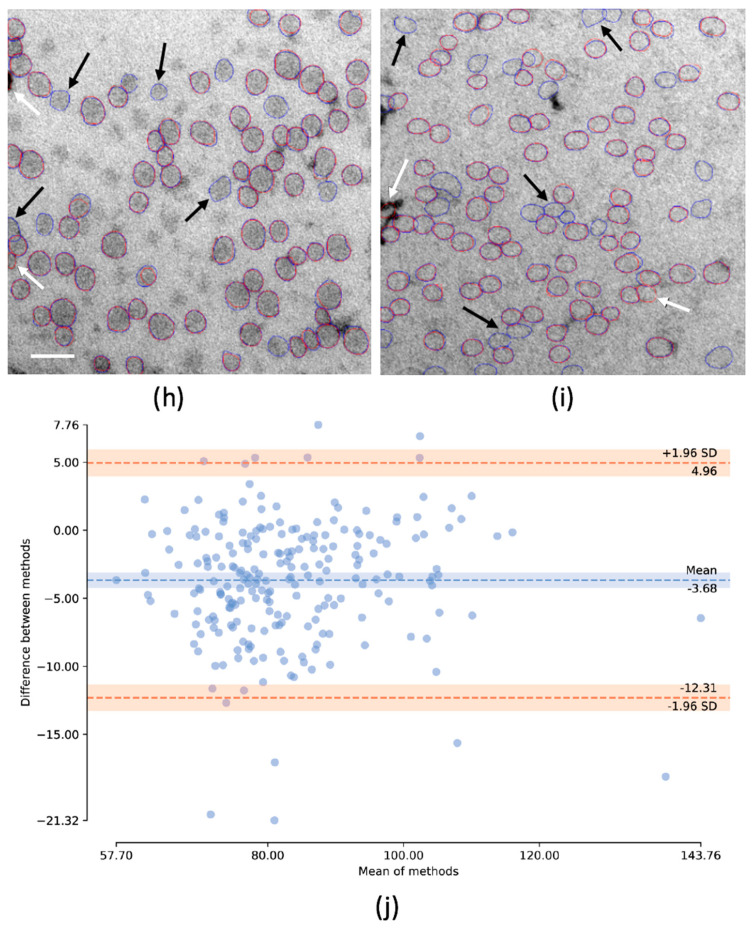
(**a**) A Mask R-CNN architecture. (Figure based on [46,47]). (**b**) A typical image from test set 1, (**c**) its manual annotation, (**d**) the Mask R-CNN output, and (**h**) the manual annotation and Mask R-CNN output overlaid. (**e**) The most challenging image from test set 1 (the image with lowest median average precision (mAP) value), (**f**) its manual annotation, (**g**) the Mask R-CNN output, and (**i**) the manual annotation and Mask R-CNN output overlaid. Arrows in (**h**) and (**i**) highlight selected differences between manual annotations and Mask R-CNN output (white arrows are false positives and black arrows false negatives). (**j**) Bland–Altman plot of agreement between particle sizes estimated automatically (Mask R-CNN) and manually (ground-truth annotation). Scale bars, 50 nm.

Table 1 gives test set 1 results for the two Mask R-CNN configurations that gave the best validation set results and the overall architecture of Mask R-CNN is illustrated in Figure 8. The model with minimum anchor scale of 16 and additional augmentation showed slightly better results by all measures except for mAP. Figure 8 illustrates this Mask R-CNN’s results on a typical test image (Figure 8b) and on the most challenging test image (Figure 8e). On the typical image, two false positives can be observed. Both are are located at the left image border and one of them covers an irregularly shaped and highly contrasted feature. Ten particles marked by the annotator were not detected; most of those had poorly contrasted contours. The more challenging image had poor contrast between particles and background, resulting in a mAP of 0.5, the lowest of any test image. Some missed detections and inaccurate instance segmentations are apparent.

Figure 8j shows a Bland–Altman plot of agreement between particle sizes estimated automatically (Mask R-CNN) and manually (ground-truth annotation). Limits of agreement were narrow, and bias was low, with a few outliers caused by large particles. However, Mask R-CNN tended to estimate particle size as smaller than the human annotator. As a simple measure of particle aggregation, the number of touching or overlapping particle pairs was computed after dilating instance segmentation masks by 1 pixel. The number of overlapping particle pairs tended to be underestimated (Table 1).

The recognition software, trained on the development set using UA/MC, was next tested on two preparations using the improved contrast offered by mixed metal methylcellulose contrasting (test set 2) and the resulting instances assessed. Both models used in Table 1 detected over 99% of particles (Table 2). The Mask R-CNN with minimum anchor scale of 32 made more false detections per 100 particles than the Mask R-CNN with minimum anchor scale of 16 (Table 2). This technique is now being used to characterize the LPs in diluted plasma samples and the results are planned to form the basis of future work on clinical samples incorporating multiplex sample deposition and automated imaging to improve throughput.

## 3. Discussion

Visualization of LPs in a preparation is an absolute requirement for characterization using EM, and here we worked to improve this [34,36]. We previously described mixed metal staining in methyl cellulose films for improving contrasting and limiting collapse of biological nanoparticles such as liposomes and nanodiscs [36]. Lipoprotein particles have sizes and protein lipid compositions that are similar to nanodiscs and, as expected, purified LDL and plasma LPs were strongly contrasted using mixed metal MC. The mixed metal MC stain shows clear advantages over contrasting in uranyl acetate MC, producing strong positive contrast at the particle periphery as well as reducing background contrast over the MC film. The improved contrast at the particle edges facilitates accurate measurement whether this is done manually, with the assistance of software such as ImageJ, or by machine learning (see below). Our studies on LDL indicate that MC film thickness is an important determinant of LP contrast and our data suggest that thinner films may help to present spheroidal particles at preferred orientations to facilitate “*en face*” measurement yielding a maximal measurement for each subclass of LP (see below, [36]). The *en face* view yields a diameter estimate of 23.74 nm (or 20.3 nm when corrected for all orientations). These values compare with the benchmark method of cryo-EM (21.4 ± 1.3 nm, height 12.1 ± 1.1 nm; average volume of 4352 nm^3^ [38]), although different preparation methods may account for the discrepancy. The advantage of cryo-EM is that it visualizes the internal details, but it is rather slow. By comparison, the positive stain method reported here is much faster and exposes the whole population for examination without substantial artefacts and will be useful for higher throughput studies. Another direct method for nanoparticle characterization is NMR and, when analyzed by ^1^H NMR spectroscopy, LDL measures 21.2 nm [48], which is comparable to our corrected value. Like other indirect measures, NMR performs calculation of lipoprotein sizes by means of the Stokes–Einstein equation [49].

A major challenge for direct image analysis of human plasma in EM is to remove noise introduced by images of plasma proteins. We found that SEC could separate physically substantial populations of the larger subtypes, of the size ranges of IDL and VLDL, from plasma proteins. This concords with previous studies [2,42,43] and more recent reports using SEC columns [50] that demonstrate a substantial yield of ApoB in protein-free SEC fractions of plasma [50]. The qEV column could have potential to separate protein from a mixed pool of lipid-rich nanoparticles prior to more refined morphological analysis (whether manual or machine assisted), providing relative and absolute numbers of different LPs. However, our analysis suggests the smallest LPs remain with the plasma proteins and a much more promising approach proved to be direct dilution of the plasma, prior to adsorption of LPs to the EM support. While undiluted plasma produces large amounts of noise from adsorbed plasma proteins, this effect is markedly reduced after dilution by three orders of magnitude, thereby allowing adsorbed LPs to become clearly visible after mixed metal MC staining.

Direct dilution provided our first quantitative EM size measurements of the LP distribution in human plasma from samples of 1 microliter or less. The visible LP particles measured from approximately 15 nm diameter and upwards, with particles clearly visualized using the mixed metal approach to a similar degree of clarity as was achieved for purified LDL. The particles can be recognized by their clear linear edge contrast, but particles with smaller dimensions, which could represent larger globular plasma proteins or HDL, were not clearly distinguished. It may be necessary, therefore, to use antibody-based approaches to identify the latter (see below). The clear positive contrast of proteins including antibodies obtained with the mixed metal MC stain could provide for low-resolution characterization of protein complex structure as an alternative to negative stain. Significantly, the size distribution of plasma LPs was shifted toward larger values compared to purified LDL. This difference could not be explained solely on the basis of larger Lp(a) particles because it remained after removal of the Lp(a) positive particles from the distribution (Figure 7). The explanation may reside in differences between plasma donors or, more likely, in methodological differences, since the LDL had been processed through a number of purification steps known to affect particle integrity and possibly the size of lipoproteins [51], whereas the plasma preparation was examined after simple dilution.

Antibodies are powerful potential tools for identifying subpopulations of LPs. This approach may be an important refinement because populations such as LDL and Lp(a) are similar in size and morphology and yet have distinctive protein compositions [16,17]. We investigated ways for visualizing antibody binding using anti-ApoB as a test case and then applied direct contrasting of bound anti-apolipoprotein(a) antibody to characterize Lp(a) particles. One other possible approach could be antibody-based aggregation of the particles on the EM support film, which quite surprisingly induced aggregation of purified LDL particles that had already adhered to the plastic support of EM grids, suggesting the particles can move on the support after absorption. This observation may be of more general utility for aggregation-based detection of molecular components in two-dimensional systems. However, because aggregation may conceal particles during measurements and will likely be inhibited in the presence of plasma proteins, this was not investigated further.

Interestingly, while immunogold had a low labelling efficiency after particle adsorption, we could label many more particles for ApoB or Lp(a) using direct labelling where antibody-sized densities appeared after application of specific antibodies. The proportion of particles labelling with ApoB- or apolipoprotein(a)-antibodies plateaued with increasing concentration, indicating saturation of the binding sites and the positive contrast of antibody molecules obtained using mixed metal MC represents a marked improvement compared to published data using negative staining [52,53]. Previously, the antibody-labelling approach was used for identification of Apo E in IDL [1], and also to identify ApoB-positive LPs, and here we used it to characterize Lp(a) particles carrying anti-apolipoprotein(a). Difficult-to-measure LP populations, such as Lp(a), can now be identified and sized. The major axis of Lp(a)-positive particles was 25.02 nm, with an equivalent sphere radius of 21.392 nm, assuming Lp(a) has the same degree of asymmetry as LDL. Lp(a) was characterised by cryo-EM as a nearly spherical particle with a radius of 21.0 nm [11], although that study used purified Lp(a). Gel electrophoresis using 29-nm beads as standards [54] measured Lp(a) at 28.27 nm and the number of kringle repeats did not seem to affect the apparent size, although there was a reduction in size by 2.23 nm after release of Lp(a) by cleavage of disulphide bonds. Our Lp(a) values were in excess of our measurements from purified LDL (by 1.613 nm), which would be consistent with the Lp(a) structure as an LDL particle extended in diameter by addition of apolipoprotein(a). Because Lp(a) particles can now be measured, it will be possible to assess whether (as is suggested for small, dense LDL) the smaller particles are more atherogenic.

In future, this type of data on human plasma LPs could be useful in developing patient-specific signatures to inform about CVD risk. LP populations such as LDL, IDL, and VLDL do not appear to generate distinct peaks on our LP distribution and, in this case, the whole size distribution or parts of it might be used to identify risk-associated populations. Another possibility would be to analyze the proportion of Lp(a) or ApoB in total particles or individual size categories. For example, we observed up to half the 20-nm-sized particles in our sample labelling for apolipoprotein(a), but this could vary from patient to patient. A further strategy might be to analyze the absolute particle numbers using our previously developed nanoparticle-counting technology [34]. One concern would be the issue of overlap and contamination with extracellular vesicles (EVs), as suggested by previous studies [2,31,32]. Extracellular vesicles range in size from 40 nm and upwards and, therefore, overlap substantially with VLDL and chylomicrons and, in particular, with remnant lipoproteins and may even cofractionate LDL [32]. In agreement with previous studies, we observed that EVs undergo collapse to produce irregular profiles [50] and comprise a tiny fraction of the total LPs. Future analysis using established markers for EVs will further validate the quantitative study of the larger-sized LPs such as VLDL.

As a step toward higher throughput, we reported results that demonstrate Mask R-CNN with transfer learning can automatically analyze EM images of lipoproteins. Models trained on a conventional UA/MC stain were then applied to the newer, mixed metal stain preparation. Better results were obtained on the mixed metal stains’ preparation even though the deep learning models had not been trained on it, likely because of improved contrast. Indeed, missed detections tended to be due to particles having poorly defined contours. False-positive detections were often located at image borders where particles were, in fact, partially visible. Some of the largest particles were not correctly identified, sometimes being divided into two smaller ones. A similar effect was observed with Mask R-CNN nuclei detection [55]. In future, use of larger, annotated image sets would be likely to improve performance. The side lengths of the anchors used in this study were powers of 2. Choosing a more varied anchor set could potentially improve performance [56]. The next steps will be to develop deep learning protocols to identify plasma nanoparticles of various sizes and differentiate minor populations of EVs from LPs or identify Lp(a) particles that are labelled with antibodies. A longer-term aim will be to multiplex sample deposition using applicators designed around technologies such as inkjet and to automate imaging prior to analysis using deep learning. Standardization and validation of the current tools combined with such high throughput would provide a fresh perspective on LP analysis and reveal indicators of CVD that could be useful to the clinician.

## 4. Materials and Methods

### 4.1. Materials and Chemicals

Methylcellulose (MC; 25 centipoise; Sigma-Aldrich, (Merck KGaA, Darmstadt, Germany; M6385)) was prepared by dispersing 2 g in 100 mL of Milli-Q water and heating to 100 °C, before cooling on ice and stirring until it dissolved. The solution was then centrifuged for 4 h at 100,000× *g* at 4 °C and supernatants removed and stored at 4 °C. Goat anti-apolipoprotein B antibody, ab98132, and sheep anti-apolipoprotein(a) (Biotin), ab27631, were from Abcam (Cambridge, UK). Protein assay kit (Pierce™ BCA Protein Assay Kit, 23225) was purchased from Thermo-Fisher Scientific, (Waltham, MA, USA). Anti-goat IgG secondary antibody was from LI-COR Biosciences, (Lincoln, NE, USA) and rabbit anti sheep Ig(H+L) antibody from Southern Biotechnology Associates Inc, (Birmingham, AL, USA). Purified LDL was obtained from EMD Millipore Corp, (Merck KGaA, Darmstadt, Germany). Human plasma was obtained from TCS Biosciences (Buckingham, UK), and stored frozen at –80 °C. Plasma was thawed rapidly in a 37 °C water bath before use. The gel filtration column was qEV single, SP2, from Izon Science (Oxford, UK).

### 4.2. Contrasting

Pioloform coated 150 or 200 mesh copper grids were floated on 5 µL droplets of plasma or LDL diluted in either Milli-Q (deionized) water or PBS on ice for 30 min before four washes in 0.7 mL droplets deionized water and contrasting in MC. Contrasting was performed essentially as described in Asadi et al. [36] and used a mixture of UA and STA in MC, referred to here as mixed metal MC contrasting or a mixture of UA and MC only (for further details see results section). Imaging was performed on a JEOL 1200 EX microscope using a Gatan Orius 200 digital camera (at University of St. Andrews, UK), a JEOL 1200 EX microscope using a Megaview II digital camera (at Dundee University, UK), or a JEOL 1400 plus microscope using a Gatan Orius 200 digital camera (at The James Hutton Institute, Invergowrie, UK). For quantification purposes, micrographs were recorded in a systematic, uniform, random-sampling pattern [57].

### 4.3. Antibody-Binding Experiments

For antibody labelling, all procedures were performed on ice in a humid chamber. Grids with attached LDL or plasma LPs (see above) were incubated on 0.1% bovine serum albumin in PBS (BSA) or 0.5% fish skin gelatin (FSG) in PBS for 10 min and then on droplets of diluted anti-ApoB or anti-apolipoprotein(a), diluted in BSA or FSG for 30 min. Following four washes on 0.7 mL droplets of PBS and four 0.7 mL droplets of Milli-Q water, mixed metal MC staining was performed. Particles were sampled and counted from micrographs by applying unbiased 2D selection sampling rules [58] to a centrally placed quadrat using an integral cell counter in Fiji (available in the “analyze” plug-in). For immunogold labelling, grids with attached purified human LDL or plasma LPs (see above) were floated on 5 µL of primary goat polyclonal anti-apolipoprotein B or sheep anti-apolipoprotein(a) antibodies diluted in 0.1% BSA or FSG. Following washes in PBS grids were incubated on intermediate rabbit anti-goat or anti-sheep antibodies diluted 1 in 500 in either BSA or FSG for 15 min. After further washes in PBS, grids were then incubated on 10-nm protein A gold (British Biocell International, Cardiff, UK) diluted 1 in 60 in 0.1% BSA or FSG, before final washes in PBS and distilled water prior to contrasting. For antibody aggregation studies, grids with attached LDL particles were incubated on 5 µL droplets of PBS or anti-ApoB antibody alone or rabbit anti-sheep (diluted 1 in 500) alone or anti-ApoB followed by PBS washes and rabbit anti-sheep (diluted 1 in 500). Then grids from each condition were washed and stained as described for the antibody-labelling procedures above. No FSG or BSA was used during these incubations. Analysis of immuno-aggregation was achieved by sampling particles and aggregates using scanning band analysis. On the live digital camera display, two geometrical features spaced by roughly half the vertical width of the field of view were used to trace out two lines during horizontal scanning. One line functioned as a forbidden line, to exclude particles/aggregates, and the other as an acceptance line [34,58]. All particles/aggregates that were completely contained between these lines or those that encountered the acceptance line were considered for analysis. For each condition, a total of approximately 100–200 events (particles/aggregates) were counted and each categorized by the number of component particles.

### 4.4. Gel Filtration

At ambient temperature, 150 µL of plasma (thawed by immersion in a water bath at 37 °C) was applied to the qEV Izon gel filtration column and eluted using PBS according to the manufacturer’s instructions. 1 mL of void volume was collected before collecting fractions of approximately 200 μL each. The fractions were stored at 4 °C. The BCA protein assay was performed as described by the manufacturer.

### 4.5. Mask R-CNN

Deep learning methods for *semantic segmentation*, the task of assigning each pixel in an image a semantic label indicating the type of object imaged at that pixel, have become popular for analyzing biological images since the success of deep convolutional neural network (CNN) architectures such as U-Net [59]. Tools for developing such models for biological microscopy applications are now available, increasing their accessibility to life scientists. For example, an ImageJ U-Net plug-in provides pretrained models for detection and analysis of specific cell types, and tools for training models for other types of cells or molecules, although it does not perform well when objects overlap [60]. The DeepCell open source library has been used to classify and segment cells [61,62]. U-net and DeepCell architectures are good candidates for nuclei analysis in fluorescence imaging [63].

Semantic segmentation assigns labels to pixels, but it does not explicitly identify individual object instances; reliably doing so from a segmentation map can be challenging when object instances touch, overlap, or occlude one another. In preliminary work, we used a symmetry detector and active contours to post-process segmentation maps in order to identify and segment LDL particle instances. While this gave reasonable results in many cases, we report here the use of a deep learning Mask R-CNN (region-based convolutional neural network) architecture [46] trained in an end-to-end manner to perform instance segmentation of LDL particles. This gave superior performance. Mask R-CNN was initially applied to computer vision datasets such as common objects in context COCO [46] but has since been used in applications such as nuclei detection and recognition of target signals in digital polymerase chain reaction fluorescence images [64].

Mask R-CNN outputs a bounding box and segmentation mask for each identified object in an image. It was proposed as an extension of a series of deep computer vision architectures for object detection and classification: R-CNN (regions with CNN features [65], Fast R-CNN [66] and Faster R-CNN [47]). This family of methods depends on mechanisms for efficiently proposing a manageable number of candidate object regions; each region of interest (RoI) is then evaluated using convolutional networks. Figure 8a outlines the architecture of a Mask R-CNN. A region proposal network (RPN) learns to propose candidate regions of different sizes and aspect ratios for object detection. This RPN is a fully convolutional network that uses *anchor* boxes that work as references. Faster R-CNN performs classification and bounding box regression on each of the regions of interest generated by the RPN. In Mask R-CNN, an additional branch is added that predicts segmentation masks for each RoI. An RoIAlign layer maintains the exact spatial location of features so that these masks are well aligned. The training loss is a combination of the losses for the class, bounding box, and mask branches of the network [46].

### 4.6. Mask R-CNN Architecture and Implementation

Our software incorporated an existing Keras/TensorFlow implementation of Mask R-CNN [67]. The architecture used had a feature pyramid network (FPN) and Residual Neural Network that is 50 layers deep (ResNet-50) backbone: *Resnet-50-FPN* in the nomenclature of [46]. A ResNet-101-FPN was also tried but was dropped in favor of ResNet-50-FPN as the former gave more false-positives on the validation set and took longer to train. The RPN anchors spanned five scales [68] but the aspect ratio was fixed so that the width and height of proposed regions were equal. Two sets of anchor scales were tried (see Table 1).

### 4.7. Transfer Learning

It helped to pretrain Mask R-CNN on larger annotated datasets for related tasks. Firstly, weights were initialized using ImageNet model weights. Secondly, the network was trained to detect nuclei on an annotated dataset of 729 microscopy images [69]. Heads only were trained for 20 epochs and then all layers for a total of 40 epochs. The number of objects detected per image was set to 400 and anchor scales used were {8, 16, 32, 64, 128} [67]. Pretraining was done on ImageNet, and then on the nuclei detection task; nuclei detection has similarities with LDL particle detection in terms of object shape.

## 5. Conclusions

It was our aim to develop an accurate method for assessment of LP populations at high resolution. This work represents initial development of appropriate tools, including contrast enhancement, antibody labelling of LP subpopulations, machine learning, and adaptation to microliter samples of patient plasma. Further progress toward higher throughput aims at multiplexed specimen deposition, automated contrasting, imaging, and calibrated particle counting. Such studies are now in progress.

## Figures and Tables

**Table 1:** Mask R-CNN results on single metal stain (test set 1).

RPN Anchor Scales (Pixels)	Augmentation Included Gaussian Noise & Sharpen?	Detection Rate (%)	False Detects per 100	mAP	Number of Overlapping Pairs (Ground Truth)
{16,32,64,128,256}	Yes	84.0	7.7	0.60	42 (63)
{32,64,128,256,512}	No	83.7	8.5	0.61	38 (63)

**Table 2:** Mask R-CNN results on mixed metal stained samples (test set 2).

Sample	RPN Anchor Scales (Pixels)	Augmentation Included Gaussian Noise & Sharpen?	Detection Rate (%)	False Detects per 100	Number of Overlapping Pairs (Ground Truth)
1	{16,32,64,128,256}	Yes	99.7	4.1	889 (895)
	{32,64,128,256,512}	No	99.3	9.4	892 (895)
2	{16,32,64,128,256}	Yes	99.9	0.6	1404 (1412)
	{32,64,128,256,512}	No	99.9	2.8	1411 (1412)

## Supplementary Materials

Supplementary materials can be found at https://www.mdpi.com/1422-0067/21/17/6373/s1.

## Abbreviations

ApoB	apolipoprotein B
BSA	0.1% bovine serum albumin in PBS
Cryo-EM	cryo-electron microscopy
CNN	convolutional neural network
CV	coefficient of variation
CVD	cardiovascular risk
EM	electron microscopy
FPN	feature pyramid network
FSG	0.5% fish skin gelatin in PBS
GPU	graphic processing unit
HDL	high-density lipoprotein
IDL	intermediate-density lipoprotein
KS	Kolmogorov-Smirnov
LDL	low-density lipoprotein
LP	lipoprotein particle
Lp(a)	lipoprotein (a)
mAP	median average precision
MC	methyl cellulose
NMR	nuclear magnetic resonance
PBS	phosphate buffered saline
R-CNN	region-based convolutional neural network
ResNet-50	residual neural network that is 50 layers deep
RLP	remnant lipoprotein
RPN	region proposed network
SD	standard deviation
sdLDL	small dense LDL
STA	sodium silicotungstate
UA	uranyl acetate
VLDL	very low-density lipoprotein
