# Supplementary material for: Developing Electron Microscopy Tools for Profiling Plasma Lipoproteins Using Methyl Cellulose Embedment, Machine Learning and Immunodetection of Apolipoprotein B and Apolipoprotein(a)
Source: Int J Mol Sci. 2020 Sep 2;21(17):6373. doi: 10.3390/ijms21176373 (PMC7503711; doi:10.3390/ijms21176373)
Supplement: Supplementary file 1 [file ijms-21-06373-s001.pdf]

International Journal of
Molecular Sciences

MDPI

Supplementary

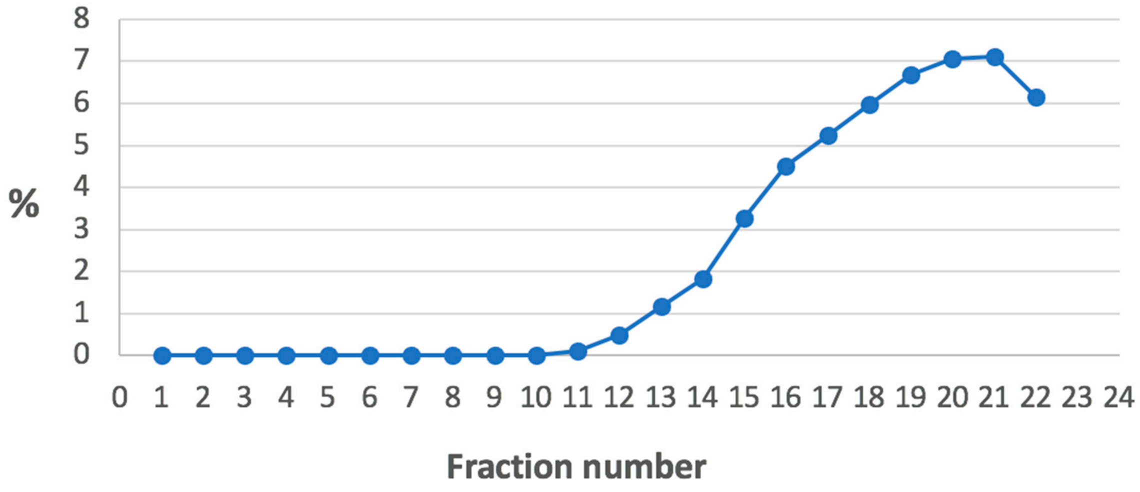

Supplementary Figure S1. Analysis of protein in fractions from the gel filtration column. Protein was assayed using the BCA method and expressed as a percentage of total protein in the plasma sample. See Materials and Methods for details.

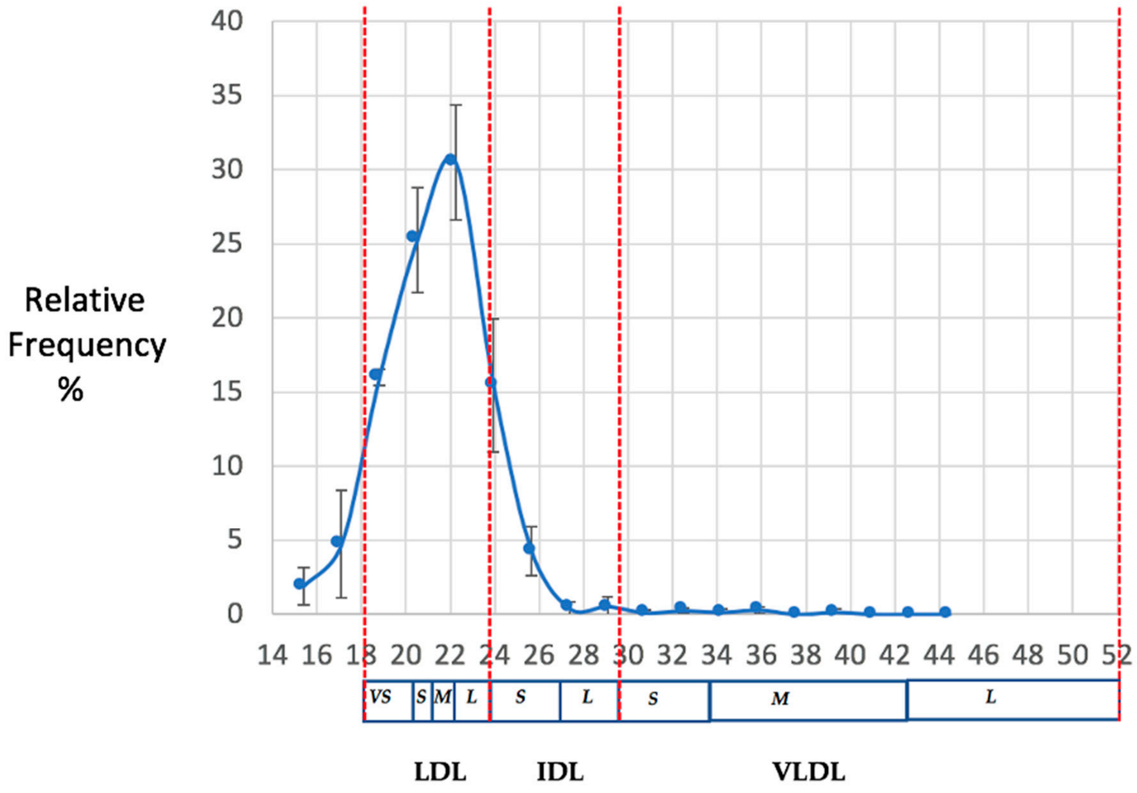

Supplementary Figure S2. Mean size distribution of plasma LPs after correcting for oblate spheroidal shape (based on LDL size ratio observed from cryo-electron microscopy [38]**)**. For comparison size ranges of lipoprotein categories from reference [10] are illustrated below the x axis: LDL very small (LDL IV, 18.0 –20.17nm), LDL small (LDL III, 20.17–21.1nm), LDL medium (LDL II, 21.1–21.99 nm) and LDL large (LDL I, 21.99 –23.8 nm); IDL IDL small (IDL2, 23.8 –26.82 nm) and IDL large (IDL1,

26.82–29.6nm) and VLDL small (29.6 –33.5 nm), medium (33.5–42.4 nm), and large (42.4 –52.0nm). For clarity the x axis label (Particle size (nm)) has been omitted.

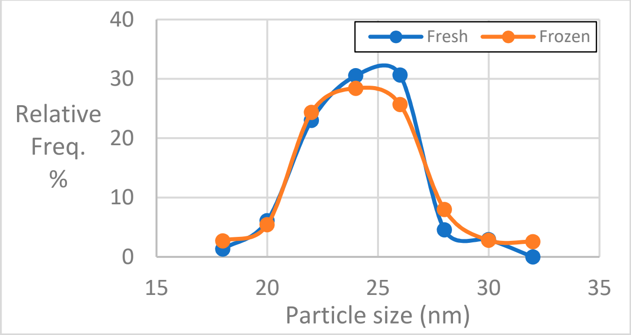

Supplementary Figure S3. Comparison of Lp(a) particles sizes in freeze-thawed (frozen) and unfrozen (fresh) plasma. Horizontal calliper distance was measured as described in Materials and Methods using ImageJ. Chi square = 1.498, df 2, $p > 0.1$. KS test**:** D is 0.095; $p = 0.895$. n = 74 (frozen) and 65 (fresh).
